# Association between vancomycin pharmacokinetic/pharmacodynamic parameters, patient characteristics, and mortality in patients with bacteremia caused by vancomycin-susceptible *Enterococcus faecium*: a single-center retrospective study

**DOI:** 10.1186/s40780-019-0138-2

**Published:** 2019-05-16

**Authors:** Ichiro Nakakura, Kota Sakakura, Kaori Imanishi, Rumi Sako, Kunio Yamazaki

**Affiliations:** Department of Pharmacy, National Hospital Organization Osaka National Hospital, 2-1-14 Hoenzaka, Chuo-ku, Osaka, 540-0006 Japan

**Keywords:** Vancomycin, Pharmacokinetics, Pharmacodynamics, *Enterococcus faecium*, Bacteremia, Prognosis, Mortality

## Abstract

**Background:**

Vancomycin is commonly used to treat *Enterococcus faecium* (*E. faecium*) bacteremia. However, there are very few studies on the association between the trough concentration, area under the curve from 0 to 24 h /minimum inhibitory concentration (AUC_24_/MIC) ratio, and the therapeutic effect of vancomycin on *E. faecium* bacteremia. This study aimed to investigate the associations between vancomycin pharmacokinetic/pharmacodynamic parameters, patient characteristics, and mortality in patients with *E. faecium* bacteremia*.*

**Methods:**

This retrospective study included patients with *E. faecium* bacteremia who received vancomycin between April 2012 and February 2018 at a single acute care hospital in Japan. Patients who received renal replacement therapy (hemodialysis or continuous hemodiafiltration), had an unmeasured serum vancomycin concentration, with unmeasured laboratory values, or received other antibiotics for treating *E. faecium* bacteremia were excluded from the study. The bivariate associations between 30-day all-cause mortality and patient characteristics were assessed.

**Results:**

Among 87 patients diagnosed with *E. faecium* bacteremia, 45 were included in the final analysis. Of these, 12 (26.7%) died within 30 days of the diagnosis. The vancomycin trough concentration was higher in the 30-day all-cause mortality patients than in the survival patients (20.5 vs. 14.6 μg/mL; *P* = 0.022). There was no significant difference in the proportion of patients with a vancomycin AUC_24_/MIC ≤389 between the groups. The 30-day all-cause mortality patients showed a higher Charlson Comorbidity Index (CCI) and Sequential Organ Failure Assessment score at the first measurement of the vancomycin trough concentration than the survival patients. The same finding was observed among patients with a high CCI score (≥5 points).

**Conclusions:**

Whereas the vancomycin trough concentration and AUC_24_/MIC ratio were not associated with mortality in patients with *E. faecium* bacteremia, disease severity was associated with mortality in these patients.

## Background

*Enterococcus faecium* (*E. faecium*) is commonly found among the commensal microflora in the human gut and mainly causes urinary tract infections, wound infections, endocarditis, and bacteremia [1]. Vancomycin-resistant *E. faecium* is a frequent isolate in the USA [2] but an infrequent one in Japan and Europe [2, 3].

Vancomycin is generally used for treating infections caused by vancomycin-susceptible *E. faecium* [4]. Previous studies have determined the appropriate target trough concentrations or area under the curve (AUC)/ minimum inhibitory concentration (MIC) ratios of vancomycin for treating methicillin-resistant *Staphylococcus aureus* (MRSA) and reported the association between the vancomycin trough concentration or AUC/MIC ratio and vancomycin’s therapeutic effect against MRSA bacteremia. Accordingly, a trough concentration of 10–20 μg/mL and an AUC/MIC ≥400 was recommended in the guidelines for therapeutic drug monitoring of vancomycin [5, 6].

Further, a recent report showed that an AUC/MIC ≥389 was associated with reduced mortality for various *Enterococcus* bacteremias [7]; however, few studies on the association between the vancomycin trough concentration or AUC/MIC ratio and the therapeutic effect against only *E. faecium* bacteremia have been conducted.

In this study, we investigated the associations between vancomycin pharmacokinetic/pharmacodynamic parameters, patient characteristics, and mortality in patients with *E. faecium* bacteremia*.*

## Methods

### Study design and setting

We performed a retrospective observational study. The study was conducted between April 1, 2012 and February 28, 2018 at a single 694-bed acute care hospital in Osaka, Japan.

### Participants and data collection

The study included participants treated with vancomycin (VANCOMYCIN HYDROCHLORIDE for I.V. Infusion 0.5 g ⌈MEEK⌋, Meiji Seika Pharma Co., Ltd., Tokyo, Japan) for *E. faecium* bacteremia. Only the first episode of *E. faecium* bacteremia was considered. Patients were followed up until discharge or in-hospital death within 30 days after diagnosis. Patients with polymicrobial bacteremia were only included in the analysis if they had received antibiotics that were active in vitro against the other coinfection pathogens.

Vancomycin was prescribed on the basis of the hospital protocol (Table 1) by physicians.

**Table 1:** Protocol for vancomycin administration

Creatinine clearance (mL/min)	Initial dose (mg/kg)	Maintenance dose (mg/kg)	Interval
≥70	25–30 (Loading dose)	15–20	8 or 12 h
50–70		10–15	12 h
30–49		10–15	24 h
< 30		7.5–12.5	24 h

The following patient data were extracted from medical records using predesigned forms: demographic characteristics (body weight, height, age, sex, underlying diseases, and admission to the intensive care unit), diagnosis of infectious diseases and source(s) of infection(s), clinical laboratory data (alanine aminotransferase, alkaline phosphatase, serum creatinine, and total bilirubin levels; platelet and white blood cell counts), Glasgow Coma Scale, ratio of partial pressure of arterial oxygen to fraction of inspired oxygen (PaO_2_/FiO_2_), urine output, 30-day all-cause mortality, administration dose of vancomycin, and initial trough concentration of vancomycin at the time of steady-state (before the fourth or more administration of vancomycin), as recommended by the guidelines [5, 6].

Patients were excluded from the study if they received renal replacement therapy (hemodialysis or continuous hemodiafiltration), had an unmeasured serum vancomycin concentration, had any unmeasured laboratory values as mentioned above, or received antibiotics other than vancomycin for the treatment of *E. faecium* bacteremia.

### Microbiology

Species identification and susceptibility tests were performed in the clinical laboratory using an automated microdilution method (BD Phoenix™ Automated Microbiology System; Becton, Dickinson and Co., Tokyo, Japan). The pathogens were considered resistant to ampicillin or vancomycin if they exceeded the Clinical and Laboratory Standards Institute breakpoints (MIC of ≥16 μg/mL for ampicillin and ≥ 32 μg/mL for vancomycin).

### Definitions

The onset of bacteremia was defined as the date of the collection of the first blood culture that yielded *E. faecium*. The suspected source of bacteremia was identified using information from the medical records.

The Charlson Comorbidity Index (CCI), Sequential Organ Failure Assessment (SOFA) score, augmented renal clearance (ARC) risk score, and estimated creatinine clearance (CLcr) were calculated as described previously [8–11]. CLcr was evaluated at the time of blood culture collection and the measurement of the initial trough concentration of vancomycin.

The SOFA score was calculated at the onset of *E. faecium* bacteremia and at the first measurement of the vancomycin trough concentration; furthermore, the difference between the two scores was calculated.

Acute kidney injury was evaluated using the Kidney Disease Improving Global Outcomes (KDIGO) criteria [12].

Treatment administered before the susceptibility results were obtained was defined as empirical therapy, whereas antimicrobial therapy given after the susceptibility data had become available was defined as definitive therapy. Treatment regimens were classified as monotherapy (treatment with 1 in vitro active agent) or combination therapy (treatment with ≥2 in vitro active agents for isolated pathogens from blood culture).

The AUC from 0 to 24 h (AUC_24_) of vancomycin was calculated through the Bayesian approach using therapeutic drug monitoring software (Vancomycin MEEK TDM analysis software Ver 3.0; Meiji Seika Pharma Co., Ltd., Tokyo, Japan). In addition, the AUC_24_/MIC ratio was calculated using the AUC_24_ calculated for each case and the MIC obtained from microbiology test results. However, when the MIC was ≤0.5 μg/mL, the AUC_24_/MIC ratio was calculated with an MIC of 0.5 μg/mL.

### Endpoints

The primary endpoint of the study was the association between the vancomycin trough concentration or the AUC_24_/MIC ratio of ≤389 and mortality in patients with *E. faecium* bacteremia. Secondary endpoints included the factors associated with all-cause mortality; these were compared between patients in the 30-day all-cause mortality and survival groups. Moreover, we performed stratified analyses by patients with acute kidney injury on pharmacokinetics of vancomycin and subgroup analysis of patients with a high CCI score (≥5 points).

### Statistical analysis

Data were analyzed using JMP® 9.0.2 (SAS Institute Inc., Cary, NC, USA). Bivariate associations between the binary outcomes of 30-day all-cause mortality and patient characteristics were assessed using the Fisher’s exact test for categorical variables and the Mann-Whitney U test for continuous variables. All statistical analyses were conducted at a significance level of 0.05.

## Results

**Fig. 1:**
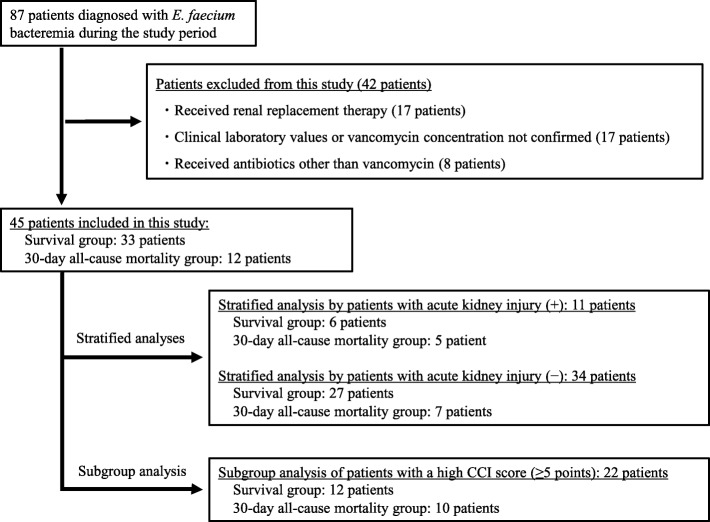
Flowchart of patient selection. *E. faecium*, *Enterococcus faecium*; CCI, Charlson comorbidity index

The patient characteristics are presented in Table 2. Among 87 patients diagnosed with *E. faecium* bacteremia during the study period, 45 patients (27 [60.0%] men and 18 [40.0%] women) were analyzed (Fig. 1). A total of 12 of the 45 patients (26.7%) died within 30 days after *E. faecium* bacteremia was diagnosed and were classified as the ‘30-day all-cause mortality group’. The 33 surviving patients were classified as the ‘survival group.’

**Table 2:** Patient and microbiological characteristics

Characteristic	All patients (*N* = 45)	30-day all-cause mortality group (*N* = 12)	Survival group (*N* = 33)	*P*-value^‡^
Sex (men: women)	27:18	8:4	19:14	0.74 ^i)^
Age (years) ^a^	73 (64–83)	71 (63–79)	75 (63–84)	0.38 ^ii)^
Height (cm)^a^	158.0 (151.8–166.0)	159.2 (153.5–169.4)	156.7 (150.3–165.3)	0.24 ^ii)^
Body weight (kg)^a^	51.0 (42.8–56.9)	56.5 (48.6–68.6)	48.8 (41.5–54.7)	0.012 ^ii)^
Number of ICU admissions	12 (26.7%)	3 (25%)	9 (27.3%)	0.11 ^i)^
ID specialist interventions	27 (60.0%)	6 (50.0%)	21 (63.6%)	0.50 ^i)^
Monotherapy (number of patients)	45	12	33	1.00 ^i)^
ALT (IU/L) ^a^	30 (16–55)	25 (13–44)	33 (16–64)	0.29 ^ii)^
ALP (IU/L, *N* = 40)^a^	544 (302–1245)	557 (306–822, *N* = 10)	545 (286–1467, *N* = 30)	0.53 ^ii)^
T–Bil (mg/dL)^a^	0.9 (0.5–3.1)	1.0 (0.5–2.8)	0.8 (0.5–3.3)	0.63 ^ii)^
WBC (× 10^3^/μL) ^a^	8.5 (4.9–12.3)	12.5 (7.0–23.8)	7.4 (4.8–11.3)	0.047 ^ii)^
PLT (× 10^3^/μL)^a^	176.0 (101.5–269.5)	101.5 (86.0–15)	237.0 (127.0–303.0)	0.0056 ^ii)^
SCr (mg/dL)^a^	0.81 (0.66–1.00)	0.79 (0.65–0.96)	0.83 (0.66–1.01)	0.75 ^ii)^
eGFR (mL/min/1.73 m^2^)^a^	67.0 (52.0–78.0)	71.0 (51.3–78.0)	66.0 (52.0–77.0)	0.69 ^ii)^
CLcr (mL/min)^ab^	53.6 (39.4–78.1)	61.7 (51.4–89.1)	48.9 (36.7–73.4)	0.10 ^ii)^
ARC risk score^a^	1 (1–1)	1 (0–1)	1 (1–1)	0.09 ^ii)^
Acute kidney injury patients^c^	11 (24.4%)	5 (41.7%)	6 (18.2%)	0.11 ^i)^
CCI^a^	4(2–6)	6 (5–7)	3 (2–6)	0.0081 ^ii)^
SOFA score (a)^a^	2 (0–4)	3 (1–6)	2 (0–3)	0.060 ^ii)^
SOFA score (b)^a^	2 (0–5)	6 (4–8)	2 (0–2)	< 0.0001 ^ii)^
SOFA score (b)-(a)^a^	0 (0–1)	3 (0–3)	0 (−1–0)	< 0.0001 ^ii)^
Suspected origin of infection				
Catheter-related infection	4	3	1	N.A
Biliary tract infection	22	5	17	
Urinary tract infection	5	2	3	
Intra–abdominal infection	5	0	5	
Unknown	9	2	7	
MIC of vancomycin for *Enterococcus faecium*				
≤ 0.5 μg/mL	14	4 (33.3%)	10 (30.3%)	NA
1.0 μg/mL	29	7 (58.3%)	22 (66.7%)	
2.0 μg/mL	1	0	1 (3.0%)	
4.0 μg/mL	1	1 (8.3%)	0	
Positive polymicrobial blood culture	15	3	12	0.72 ^i)^

SOFA score (a): at the onset of *E. faecium* bacteremia

SOFA score (b): at the first measurement of the vancomycin trough concentration

*ALT* alanine aminotransferase, *ALP* alkaline phosphatase, *ARC* augmented renal clearance, *CCI* Charlson Comorbidity Index, *CLcr* estimated creatinine clearance, *eGFR* estimated glomerular filtration rate, *ICU* intensive care unit, *ID* infectious disease, *MIC* minimum inhibitory concentration, *NA* not available, *PLT* platelet count, *SCr* serum creatinine level, *SOFA* Sequential Organ Failure Assessment, *T-Bil* total bilirubin, *WBC* white blood cell count

i) Fisher’s exact test, ii) Mann-Whitney U test

^a^Expressed as median (interquartile range, IQR)

^b^Calculated by the Cockcroft-Gault equation

^c^Patients with acute kidney injury evaluated at the first measurement of the vancomycin trough concentration

^‡^The *P*-value was calculated by processing the comparison between the 30-day all-cause mortality group and the survival group by an appropriate statistical analysis

The median CCI was significantly different between the two groups (6 points [IQR, 5–7 points] in the 30-day all-cause mortality group vs. 3 points [IQR, 2–6 points] in the survival group; *P* = 0.0081). The median SOFA score at the onset of *E. faecium* bacteremia was not significantly different between the groups (3 points [IQR, 1–6 points] in the 30-day all-cause mortality group vs. 2 points [IQR, 0–3 points] in the survival group; *P* = 0.060). In contrast, the median SOFA score at the first measurement of the vancomycin trough concentration was significantly different between the groups (6 points [IQR, 4–8 points] in the 30-day all-cause mortality group vs. 2 points [IQR, 0–2 points] in the survival group; *P* < 0.0001).

The suspected origins of the infections are summarized in Table 2. In the overall study population (*n* = 45 patients), the most common probable source of bacteremia was biliary tract infection in 22 patients (5 patients in the 30-day all-cause mortality group and 17 patients in the survival group); cases of endocarditis and meningitis were not included.

The pharmacokinetics and administration of vancomycin are shown in Table 3. There was a significant difference in the vancomycin trough concentration between the two groups (20.5 μg/mL [IQR, 13.4–26.7 μg/mL] in the 30-day all-cause mortality group vs. 14.6 μg/mL [IQR, 9.3–19.2 μg/mL] in the survival group; *P* = 0.022). However, we identified no significant differences in the number of patients with evaluated acute kidney injury (5 of 12 patients in the 30-day all-cause mortality group vs. 6 of 33 patients in the survival group; *P* = 0.11) and in the ARC risk score (1 point [IQR, 0–1 points] in the 30-day all-cause mortality group vs. 1 point [IQR, 1–1 points] in the survival group; *P* = 0.09) between the groups (Table 2).

**Table 3:** Pharmacokinetics and administration of vancomycin

Characteristic	All patients (*N* = 45)	30-day all-cause mortality group (*N* = 12)	Survival group (*N* = 33)	*P*-value^†^
Vancomycin trough concentration (μg/mL)^a^	15 (9.7–22.3)	20.5 (13.4–26.7)	14.6 (9.3–19.2)	0.022^i)^
Frequency of vancomycin administration until the first measurement of the vancomycin trough concentration^a^	3 (3–5)	3 (3–5)	3 (3–5)	0.36^i)^
Vancomycin AUC_24_ (μg/mL·h)^a^	665 (444–898)	721 (380–860)	651 (462–952)	0.77^i)^
Vancomycin AUC_24_/MIC ratio^a^	725 (450–1186)	792 (332–1040)	707 (450–1304)	0.76^i)^
Number of cases with failure to achieve a vancomycin AUC_24_/MIC ratio ≤ 389	8 (17.8%)	3 (25.0%)	5 (15.2%)	0.66^ii)^
Duration of vancomycin administration (days)^a^	11.5 (7.0–16.5)	7 (4.0–11)	15 (10–25)	< 0.001^i)^
End of vancomycin administration due to death	8	8	0	–
Vancomycin used for empirical therapy	29 (64.4%)	9 (75.0%)	20 (60.6%)	0.49^ii)^
Duration from blood culture collection to vancomycin administration (days)^a^	3 (1–4)	1 (1–4)	3 (1–4)	0.30^i)^

*AUC*_*24*_ estimated area under the curve from 0 to 24 h, *MIC* minimum inhibitory concentration

i) Mann-Whitney U test, ii) Fisher’s exact test

^a^Expressed as medians and interquartile ranges (IQR)

^†^The *P*-value was calculated by processing the comparison between the 30-day all-cause mortality group and the survival group by an appropriate statistical analysis

The AUC_24_ (721 μg/mL·h [IQR, 380–860 μg/mL·h] in the 30-day all-cause mortality group vs. 651 μg/mL·h [IQR, 462–952 μg/mL·h] in the survival group; *P* = 0.77) and AUC_24_/MIC ratio (792 [IQR, 332–1040] in the 30-day all-cause mortality group vs. 707 [IQR, 450–1304] in the survival group; *P* = 0.76) were not significantly different between the groups. Further, 3 of 12 patients (25.0%) in the 30-day all-cause mortality group and 5 of 33 patients (15.2%) in the survival group had a vancomycin AUC_24_/MIC ratio of ≤389 (*P* = 0.66).

Stratified analyses by patients with acute kidney injury on pharmacokinetics of vancomycin are shown in Table 4. Regardless of the presence or absence of acute kidney injury, there was no significant difference between the 30-day all-cause mortality group and the survival group with respect to pharmacokinetics of vancomycin.

**Table 4:** Stratified analyses by acute kidney injury on pharmacokinetics of vancomycin

	Characteristic	30-day all-cause mortality group	Survival group	*P*-value^†^
Acute kidney injury (+)	Number of patients	5	6	–
	Vancomycin trough concentration (μg/mL)^a^	26 (22.3–33.2)	12.8 (9.1–30.5)	0.20 ^i)^
	Vancomycin AUC_24_ (μg/mL·h)^a^	859 (393–971)	794 (486–1052)	0.86 ^i)^
	Vancomycin AUC_24_/MIC ratio^a^	859 (427–971)	809 (459–1409)	0.86 ^i)^
	Number of cases with failure to achieve a vancomycin AUC_24_/MIC ratio ≤ 389	1	0	0.45 ^ii)^
Acute kidney injury (−)	Number of patients	7	27	–
	Vancomycin trough concentration (μg/mL)^a^	15.0 (12.7–21.4)	14.6 (9.3–18.2)	0.30 ^i)^
	Vancomycin AUC_24_ (μg/mL·h)^a^	498 (354–765)	645 (417–853)	0.48 ^i)^
	Vancomycin AUC_24_/MIC ratio^a^	725 (277–1531)	707 (417–1319)	0.75 ^i)^
	Number of cases with failure to achieve a vancomycin AUC_24_/MIC ratio ≤ 389	2	5	0.61 ^ii)^

*AUC*_*24*_ estimated area under the curve from 0 to 24 h, *MIC* minimum inhibitory concentration

i) Mann-Whitney U test, ii) Fisher’s exact test

^a^Expressed as medians and interquartile ranges (IQR)

^†^The *P*-value was calculated by processing the comparison between the 30-day all-cause mortality group and the survival group by an appropriate statistical analysis

No significant difference was observed in the number of days from blood culture collection to vancomycin administration (1 day [1–4 days] in the 30-day all-cause mortality group vs. 3 days [1–4 days] in the survival group; *P* = 0.30) between the two groups (Table 3).

The characteristics of *E. faecium* and positive polymicrobial blood culture results are summarized in Table 2. All 45 *E. faecium* cases were susceptible to vancomycin and resistant to ampicillin. Most strains had a vancomycin MIC value of 1 μg/mL (in 29 strains), followed by ≤0.5 μg/mL (in 14 strains). We observed no significant difference in polymicrobial bacteremia between the two groups (3 of 12 patients in the 30-day all-cause mortality group vs. 12 of 33 patients in the survival group; *P* = 0.72).

The results of the subgroup analyses in patients with a high CCI score are depicted in Table 5. There was no significant difference in the CCI score (6 points [IQR, 6–8 points] in the 30-day all-cause mortality group vs. 6 points [IQR, 6–7 points] in the survival group; *P* = 0.77) and the median SOFA score at the onset of *E. faecium* bacteremia (2 points [IQR, 1–6 points] in the 30-day all-cause mortality group vs. 1 point [IQR, 1–5 points] in the survival group; *P* = 0.40) between the groups. However, there was a significant difference in the vancomycin trough concentration between the two groups (23.2 μg/mL [IQR, 14.4–28.3 μg/mL] in the 30-day all-cause mortality group vs. 10.5 μg/mL [IQR, 8.9–15.7 μg/mL] in the survival group; *P* = 0.0069) and the median SOFA score at the first measurement of the vancomycin trough concentration (6 points [IQR, 4–7 points] in the 30-day all-cause mortality group vs. 2 point [IQR, 0–2 points] in the survival group; *P* = 0.014).

**Table 5:** Characteristics of patients with a high Charlson Comorbidity Index (≥5 points) as well as pharmacokinetics and pharmacodynamics of vancomycin

Characteristic	All patients (*N* = 22)	30-day all-cause mortality group (*N* = 10)	Survival group (*N* = 12)	*P*-value^†^
Body weight (kg)^a^	52.7 (45.7–63.0)	60.4 (46.8–71.0)	48.7 (42.6–54.3)	0.044 ^i)^
WBC (× 10^3^/μL)^a^	9.1 (4.0–13.2)	13.2 (7.4–17.4)	5.8 (3.5–11.4)	0.048 ^i)^
PLT (× 10^3^/μL)^a^	154 (97.5–206.5)	102 (76–152)	181 (125–292)	0.021 ^i)^
CCI^a^	6 (6–7)	6 (6–8)	6 (6–7)	0.77 ^i)^
SOFA score (a)	2 (1–5)	2 (1–6)	1 (1–5)	0.40 ^i)^
SOFA score (b)	3 (1–6)	6 (4–7)	2 (0–2)	0.014 ^i)^
Acute kidney injury patients^¶^	8 (36.4%)	4 (40.0%)	4 (33.3%)	1.00 ^ii)^
ARC risk score^a^	1 (0–1)	1 (0–1)	1 (0–1)	0.57 ^i)^
Pharmacokinetics and pharmacodynamics of vancomycin				
Vancomycin trough concentration (μg/mL)^a^	14.8 (9.9–25.3)	23.2 (14.4–28.3)	10.5 (8.9–15.7)	0.0069 ^i)^
Vancomycin AUC_24_ (μg/mL·h)^a^	721 (498–943)	745 (488–885)	658 (499–1065)	1.00 ^i)^
Vancomycin AUC_24_/MIC ratio^a^	826 (561–1142)	859 (663–1194)	730 (515–1242)	0.74 ^i)^
Number of cases with failure to achieve a vancomycin AUC_24_/MIC ratio ≤ 389	10 (45.5%)	5 (50.0%)	5 (41.7%)	1.00 ^ii)^

*AUC*_*24*_ estimated area under the curve from 0 to 24 h, *CCI* Charlson Comorbidity Index, *MIC* minimum inhibitory concentration, *PLT* platelet count, *SOFA* Sequential Organ Failure Assessment, *WBC* white blood cell count

i) Mann-Whitney U test, ii) Fisher’s exact test

^a^Expressed in medians and interquartile ranges (IQR)

^†^The *P*-value was calculated by processing the comparison between the 30-day all-cause mortality group and the survival group by an appropriate statistical analysis

¶Patients with acute kidney injury evaluated at the first measurement of the vancomycin trough concentration

SOFA score (a): at the onset of E. faecium bacteremia

SOFA score (b): at the first measurement of the vancomycin trough concentration

## Discussion

This study found no significant association in bacteremia cases caused by *E. faecium*, either with the AUC_24_/MIC ratio or the appropriate trough concentration of vancomycin, with mortality. Rather, the SOFA score during the measurement of the initial trough concentration of vancomycin was higher in the 30-day all-cause mortality group than in the survival group, suggesting that disease severity was associated with mortality.

The mortality rate of patients with *E. faecium* bacteremia has been reported as 25.0–34.6% [13–16], and is similar to the 30-day all-cause mortality rate in our study (26.7%) that included patients with *E. faecium* bacteremia treated with vancomycin only.

Previous studies showed the following risk factors for mortality in patients with *E. faecium* bacteremia: current corticosteroid use, intensive care unit admission [13], a high CCI score, pulmonary infection, and nosocomial infection [14]. A recent retrospective study reported reduced mortality in patients with enterococcal (including *E. faecium*) bacteremia with a vancomycin AUC/MIC_E-test_ ratio ≥ 389 [7]. However, in that study [7], vancomycin was administered according to hospital protocol, and the median blood concentration of vancomycin was lower than the blood concentration recommended for *Staphylococcus* bacteremia, in particular, in patients who died; thus, we hypothesize that the vancomycin dose was inadequate.

In our study, among all patients, the CCI was significantly higher in the 30-day all-cause mortality than in the survival group. However, in the subgroup analyses of patients with a high CCI score, prognostic factors at baseline such as the SOFA score and CCI were not significantly different between the 30-day all-cause mortality and survival groups. In our study, vancomycin was also administered according to hospital protocol; however, the median blood concentration of vancomycin in both groups achieved or exceeded the recommended concentration for the treatment of MRSA infection according to the guidelines for therapeutic drug monitoring of vancomycin [5, 6]. Furthermore, the effect of the pharmacokinetics of vancomycin, such as the frequency of acute kidney injury and ARC risk score, were not significantly different between the groups. However, the median blood concentration of vancomycin was significantly higher in the 30-day all-cause mortality group than in the survival group in the total study population and in patients with a high CCI score. In contrast, the proportion of patients achieving AUC_24_/MIC ratios ≤389 (for *Enterococci*) was not different between the two groups, both in the entire study population and in the subgroup of patients with a high CCI score. Therefore, our results suggest that the trough blood concentration and the AUC_24_/MIC ratio of vancomycin that were recommended for MRSA treatment were not associated with mortality in patients with *E. faecium* bacteremia.

One of the reasons may be related to the antimicrobial activity of vancomycin against Gram-positive bacteria. Vancomycin has bactericidal activity against Gram-positive pathogens (including Staphylococci and Streptococci) and bacteriostatic activity against enterococci [17]. Generally, bacteriostatic antibiotics require phagocytic cells to definitely eliminate bacteria and are therefore considered less effective without an efficient immune response [18]. Accordingly, a previous study showed the importance of using bactericidal antibiotics in the treatment of endocarditis or meningitis [19]. Another study concluded that the categorization of antibiotics as bacteriostatic and bactericidal is likely not relevant in clinical practice if these antibiotics are used for abdominal infections, skin and soft tissue infections, and pneumonia [18]. In conclusion, the difference in the therapeutic effect on bacteremia between bactericidal and bacteriostatic antibiotics remains unclear. In our study, no cases of endocarditis and meningitis were included.

The cases in the 30-day all-cause mortality group were more severe illness than those of the survival group at the first measurement of vancomycin trough concentration, and thus, the patients were considered to have lower immunity. Therefore, we considered that a high serum concentration of vancomycin does not necessarily increase the drug’s therapeutic effect against enterococcal infection.

In this study, we considered disease severity and patient characteristics as risk factors for mortality in patients with *E. faecium* bacteremia. In particular, patients in the 30-day all-cause mortality group had higher SOFA scores at the first measurement of the vancomycin trough concentration than those in the survival group, suggesting that the 30-day all-cause mortality group had higher disease severity.

Our study has some limitations. This was an observational study conducted at a single center, and the number of cases was limited. Unlike in a recent retrospective study [7], in our study, the MIC was measured using an automated microdilution method; moreover, a different calculation method of the AUC_24_, the Bayesian method that used one-point trough concentration and the population data of *Staphylococcus aureus* infections, was used. Therefore, the pharmacokinetics of acute kidney injury patients was not included in the population data, so AUC may be more inaccurate for these cases. Moreover, it is not possible simply to compare the AUC or AUC/MIC between our study and that of a recent retrospective study [7]. In both, in patients who died and those who survived, the blood concentration of vancomycin was within the effective treatment range for *Staphylococcus aureus* in many cases. As our study did not include enough cases with low AUC_24_/MIC ratios, we could not verify that a low AUC_24_/MIC ratio leads to a poor prognosis. In addition, in our case, CCI was higher in the 30-day all-cause mortality cases than in survival cases, and CCI ≤ 5 points patients in the 30-day all-cause mortality cases group were very few. Therefore, we consider that there is a need for further study in cases with lower severity.

## Conclusions

Our results suggest that the trough blood concentration and the AUC_24_/MIC ratio of vancomycin that are recommended for MRSA treatment were not associated with mortality in patients with *E. faecium* bacteremia. These conclusions are different from those of a recent retrospective study [7]. For this reason, further studies (in particular, prospective trials) are needed to clarify the association between the vancomycin trough concentration or the vancomycin AUC and mortality in patients with *E. faecium* bacteremia.

## Abbreviations

ARC: Augmented renal clearance
AUC: Area under the curve
AUC_24_: Estimated AUC from 0 to 24 h
CCI: Charlson Comorbidity Index
CLcr: Estimated creatinine clearance
*E. faecium*: *Enterococcus faecium*
KDIGO: Kidney Disease Improving Global Outcomes
MIC: Minimum inhibitory concentration
MRSA: Methicillin-resistant *Staphylococcus aureus*
PaO2/FiO2: Ratio of partial pressure of arterial oxygen to fraction of inspired oxygen
SOFA: Sequential Organ Failure Assessment
